# High Rate Dynamic Monitoring with Fabry–Perot Interferometric Sensors: An Alternative Interrogation Technique Targeting Biomedical Applications

**DOI:** 10.3390/s19214744

**Published:** 2019-10-31

**Authors:** M. Fátima Domingues, Cátia Tavares, Nélia Alberto, Ayman Radwan, Paulo André, Paulo Antunes

**Affiliations:** 1 Instituto de Telecomunicações, Campus Universitário de Santiago, 3810-193 Aveiro, Portugal; nelia@ua.pt (N.A.); aradwan@av.it.pt (A.R.); pantunes@ua.pt (P.A.); 2 Department of Physics & I3N, University of Aveiro, 3810-193 Aveiro, Portugal; catia.tavares@ua.pt; 3 Department of Electrical and Computer Engineering and Instituto de Telecomunicações, Instituto Superior Técnico, University of Lisbon, 1049-001 Lisbon, Portugal; paulo.andre@tecnico.ulisboa.pt

**Keywords:** optical fiber, Fabry–Perot interferometric sensors, high rate interrogation architecture, biomedical applications

## Abstract

Fabry–Perot interferometric (FPI) sensors are an accurate and well-established sensing technology that are used to monitor a wide range of parameters such as strain, temperature, and refractive index, among many others. Nevertheless, due to the limited number and high cost of existing interrogation techniques for FPIs, its use is often restricted to discrete measurements, not being so explored for dynamic applications. The development of an alternative interrogation technique for a high rate of acquisition may propel this type of sensor into less explored fields such as dynamic biomedical applications. In this work, we present the theoretical and experimental analyses of an FPI sensing architecture by using an alternative high rate dynamic acquisition methodology, based on frequency to amplitude conversion, where the FPI spectral shift is detuned by the convolution of the optical light source with the FPI interference pattern. The good agreement between the theoretical and experimental results verified the reliability of the proposed methodology. Moreover, preliminary results show that the developed sensing architecture can be a suitable solution to monitor biomedical parameters such as the carotid pulse wave.

## 1. Introduction

In-line optical fiber interferometers are, nowadays, an accurate, reliable, and high resolution sensing solution for applications in fields such as biomedicine and healthcare. Their prospective applications include the monitoring of parameters such as pressure, strain, refractive index, temperature, humidity, and magnetic field, among others [1,2,3]. Optical fiber Fabry–Perot interferometric (FPI) sensors, particular in-line FPIs, have advantageous characteristics to all optical fiber sensors, namely: immunity to electromagnetic interference, high resolution, electronically safe, fast response (dependent on the interrogation device), and compact size. Moreover, due to the vast research and resulting advances in their production methodology, mostly related to fusion splicing methods, in-line FPIs are also becoming a cost-effective solution for sensing applications [1,3,4,5]. Other production techniques, although effective, may require the use of expensive devices or complex positioning systems such as the micromachining or the use of femtosecond lasers [3].

An FPI sensing device is typically formed by two parallel light-reflecting surfaces, separated by a given length. When a broadband light signal is injected in the optical fiber at the FPI location, an interference pattern is created, as a consequence of the optical reflections within the FPI micro-cavity and its interaction with the injected optical signal. Considering the interference generated at the FPI, the intensity, *I*, of the spectrum modulated at the FPI micro-cavity can be given by:

(1) $$I=\;I_{1}+I_{2}+cos\left(\delta \right)\sqrt{I_{1}I_{2}}$$

where *I_1_* and *I_2_* are the intensities of the reflected optical signal in the two mirrored surfaces of the FPI [6]. *δ* represents the round trip optical phase difference between two adjacent signals at a normal angle of incidence, and can be expressed as:

(2) $$\delta =\;\frac{4\pi nL}{\lambda }$$

where *L* is the micro-cavity length; *n* corresponds to the micro-cavity’s material refractive index (assumed as 1, considering the air refractive index); and *λ* is the optical signal wavelength [6].

According to Equations (1) and (2), the FPI modulated spectra maximums occur for:

(3) $$\frac{4\pi nL}{\lambda_{m}}=2m\pi$$

where *m* is an integer and *λ_m_* is the initial central wavelength of the *m^th^* order spectrum maximum. The wavelength difference, corresponding to two consecutive maximums or two consecutive minimums of the FPI reflected transfer function, is known as the free spectral range (*FSR*). Therefore, *FSR* corresponds to the range of wavelengths, where two consecutive orders, *m* and *m + 1*, do not overlap, and can be translated by [6]:

(4) $$FSR=\frac{\lambda^{2}}{2nL}$$

Most often, the analysis of the signal, modulated at the FPI location, is based on the reflected spectrum, in terms of *FSR* variation or wavelength shift, considering the discrete acquisition of the spectra by means of an optical interrogator, or an apparatus comprising a broadband optical source and an optical spectrum analyzer (OSA) [4,5]. Other reported approaches have used a light emitting diode (LED) as the optical source, nevertheless the set ups, presented in the referenced studies, require at least two set of sensors in the overall architecture and complex demodulation techniques [7,8].

The wide application of optical fiber FPI sensing devices depends greatly on the efficiency, the ease of use, and the reduced financial cost of the interrogation systems, required to analyze the response of these sensors. Furthermore, the application of in-line optical fiber FPIs in dynamic applications such as accelerometers, heart, or breath rate, or even human body range of motion monitoring, is still limited by the lack of cost-effective solutions for high rate acquisition monitoring devices that this type of sensing involves. The existing high rate acquisition optical devices require a high financial investment and the posterior extensive data processing for spectral analysis.

In this work, we evaluated the use of a cost-effective FPI production and interrogation technique toward the application of this type of sensing element for dynamic and biomedical applications. The proposed architecture is an efficient and financially sustainable alternative to the FPI high rate interrogation systems. The solution proposed is composed of the sensing element and an alternative acquisition methodology, based on frequency to amplitude conversion, where the FPI spectrum is detuned by the convolution between a narrow band optical source and its modulation at the FPI micro-cavity. Adding to the low cost and complexity requirements of the proposed interrogation set up, the in-line FPI micro-cavity was produced based on the recycling of optical fiber, previously damaged by the catastrophic fiber fuse effect by splicing techniques [4,5].

The rest of this paper is organized as follows. Section 2 presents the FPI production methodology and the proposed interrogation architecture; in Section 3, the designed architecture is theoretically and experimentally analyzed, while in Section 4, we show the preliminary results obtained for the carotid pulse wave monitoring using the proposed overall architecture. The conclusions and future applications are considered in Section 5.

## 2. Fabry-Perot Interferometric Micro-Cavity Production and Interrogation Architecture

**Figure 1:**
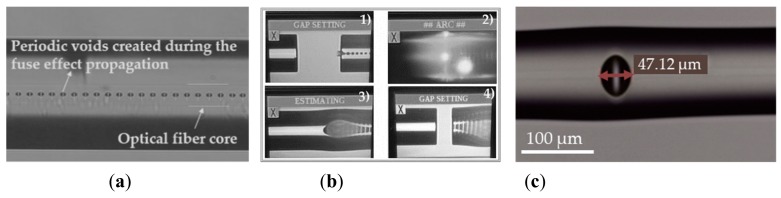
(**a**) Microscopy of an optical fiber containing the voids generated by the fuse effect; (**b**) Procedure used to fabricate the Fabry-Perot interferometric (FPI) micro-cavity by recycling the damaged optical fiber; (**c**) Microscopy image of the produced in-line FPI micro-cavity used for the experimental characterization.

The in-line FPI micro-cavity, presented in this work, was produced based on the recycling of optical fiber previously damaged by the catastrophic fuse effect, as described in [4,5]. After the fuse effect propagation, the optical fiber becomes permanently damaged. This damage appears in the form of periodic voids in the optical fiber core, as depicted in Figure 1a, which blocks the optical signal transmission [9].

The production of the FPI micro-cavity is achieved by splicing the damaged fiber to a standard single mode fiber (SMF) (Figure 1(b.1,1b.2)). This first step leads to a bigger void in the boundary between the SMF fiber and the damaged fiber, as shown in Figure 1(b.3). In order to produce a single in-line micro-cavity, the fiber is then cleaved again a few micrometers away from the first splicing point and spliced to another SMF fiber (Figure 1(b.4)). The final micro-cavity produced is depicted in Figure 1c.

The interrogation of the FPI sensors is most often based on the analysis of the reflected transfer function in the FPI resonant micro-cavity. Such analysis restrains the application of this type of sensors, both economically and logistically, since they require both bulky and expensive equipment such as the typical OSA, broadband light sources, or interrogator devices. Moreover, such interrogation techniques render them less attractive for high rate acquisition applications, namely accelerometers, or for health applications (heart rate monitoring, gait analysis, …).

**Figure 2:**
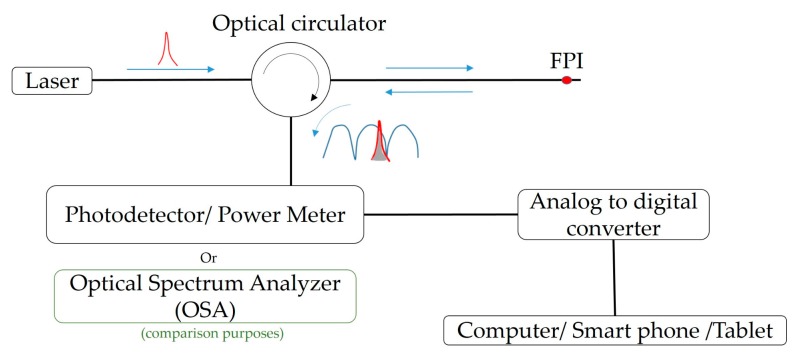
Proposed FPI interrogation architecture for high rate dynamic applications.

To mitigate such drawbacks of the FPI sensors, and envisioning their use for dynamic monitoring applications, we propose an efficient high rate acquisition interrogation alternative that is compact and financially sustainable, based on a frequency-to-amplitude conversion, where the FPI spectrum is demodulated by the optical signal interference amplitude. The proposed high rate acquisition sensing architecture comprises a narrow band optical source (FOL1405RTD-657-1509, Fitel, Tokyo, Japan), centered at 1508 nm with a full width at half maximum (FWHM) of 2.079 nm, and an optical circulator. The optical signal is launched into the circulator and modulated at the FPI micro-cavity. The integrated area, resulting from the optical source and FPI optical spectra convolution, is then monitored by a photodetector (Model D400FC, Thorlabs, Newton, NJ, USA), as shown in Figure 2. The optical signal, acquired by the photodetector, is processed using an analog to digital converter (USB6008, National Instruments, Austin, TX, USA). This, in this way, the signal monitoring can be performed using a computer, or, if considering wearable eHealth applications, the same software can be adapted for a mobile app on a smart phone or tablet.

This configuration results in a less complex apparatus compared to the one often employed with edge filtering interrogation techniques, where the signal demodulation results from the filtering of the optical signal modulated at the FPI, which requires additional optical components (sources, couplers, and optical elements) [10]. Using a narrow band optical source, no edge filtering is required. In this case, the reflected optical power amplitude, resulting from the integrated area of convolution between the FPI modulated signal and laser spectral function, will allow the interrogation of the sensor, at rates dependent only on the photodiode and acquisition interface devices specifications. 

**Figure 3:**
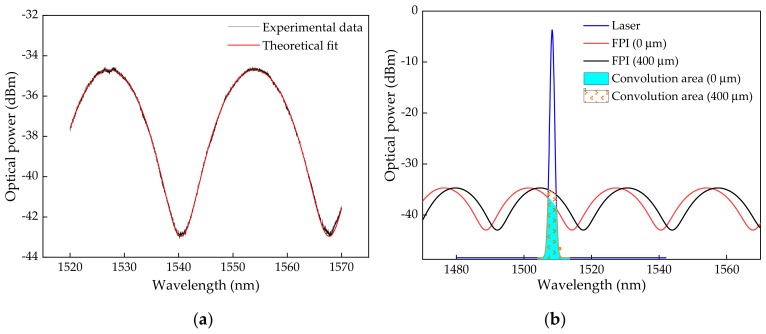
(**a**) Produced FPI transfer function and the correspondent fit to Equation (1); (**b**) Illustration of the laser and FPI convolution integrated area variation with an increased elongation.

Considering the produced micro-cavity, it presents a transfer function as depicted in Figure 3a (acquired with the interrogation device SM125, Micron Optics Inc., Atlanta, GA, USA), which can be easily fit to the Equation (1). From the fitting parameters, a micro-cavity length, *L*, of 44.07 µm was obtained, which is in accordance with the value obtained, based on the micro-cavity microscopy (47.12 μm) (Figure 1c).

In Figure 3b, two simulated spectra, based on Equation (1), are presented, for a wider wavelength range of 1470–1570 nm, considering the FPI with no deformation applied, and a second spectrum considering a deformation corresponding to an applied longitudinal elongation of 400 µm, which is translated by a positive wavelength shift. Additionally, in Figure 3b, the simulated laser spectrum is presented considering its fit to a Gaussian function, and the predicted convolution resulting from the laser modulation by the FPI for both situations of 0 µm and 400 µm of elongation.

The shift of the FPI spectrum to higher wavelengths, due to the applied deformation on the fiber, will change the total optical power reaching the photodetector, as a result of the variation of the reflected integrated area of the convolution between the laser and the FPI optical spectrum. This convolution corresponds to the area of the overlap between the FPI transfer function (*I*(*λ*)) and the laser Gaussian function *G*(*λ*). In that way, the optical power (*OP*), resulting from the convolution integrated area, can be calculated considering the convolution function given by:

(5) $$OP\left(\lambda \right)=I\left(\lambda \right).G\left(\lambda \right)\int_{\lambda_{i}}^{\lambda_{f}}I\left(t\right)G\left(\lambda -t\right)dt$$

where *t* is a variable of integration. Within the proposed architecture, the optical power resulting from the convolution integrated area, represented in blue for 0 µm and dots for 400 µm of elongation, corresponds to the total optical power acquired by the photodetector, which will increase with the increase of the applied deformation as the FPI transfer function shifts for higher wavelengths.

The modulation of the FPI transfer function will induce its wavelength shift, and a consequent variation of the resulting optical power integrated area. Therefore, by monitoring the optical power reflected at the FPI location, it is possible to monitor parameters able to modulate the in-line FPI transfer function such as strain or temperature. In the following sections, the theoretical analysis and experimental implementation of the described interrogation architecture for monitoring the produced FPI under strain variations will be presented.

## 3. Theoretical Analysis and Experimental Characterization

**Figure 4:**
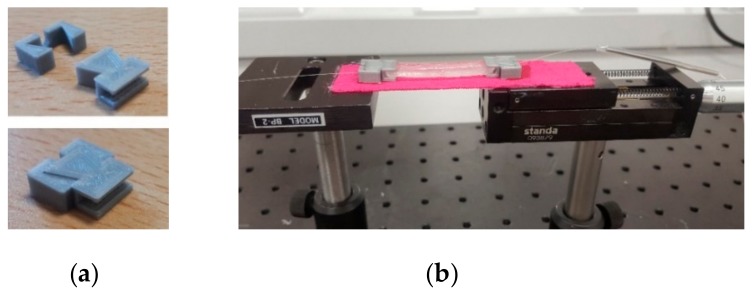
(**a**) Polylatic acid 3D printed adaptors for the sensing resin block; (**b**) Experimental set up for strain characterization with the assembled sensing block: optical fiber in-line FPI integrated in a resin structure, incorporated in the 3D adaptors and attached to the adhesive biocompatible elastic tape.

Envisioning its future application, the sensor was encapsulated in a thermal setting epoxy-resin (Liquid Lens, Leighton Buzzard, Bedforshire, UK) structure to provide the necessary robustness and resilience to the optical fiber and in-line FPI. The epoxy resin is a two-part mixture liquid thermal setting resin, which during the curing process forms a strong bond with the optical fiber. For the optical fiber encapsulation, the section of the fiber containing the FPI sensor was placed in a polylatic acid (PLA) 3D printed mold with the internal dimensions of 4.0 × 1.0 × 0.3 cm. The mold was then filled with the epoxy resin and given a cure time of 24 h to grant the full setting of the resin. Additionally, to facilitate the sensor’s re-use and re-application, a set of PLA 3D printed adaptors (Figure 4a) was incorporated at both extremities of the sensing block. The resin block containing the optical fiber was firmly attached to the adaptors depicted in Figure 4a, and the The final structure was fixed () to an adhesive biocompatible elastic tape, Figure 4b, both with high bond acrylate glue, to promote its ease of application for human body motion monitoring or wearable eHealth architectures. The whole structure was also glued to the translation stage apparatus, with the anchorage points set at the adaptors location (6 cm apart).

When the elastic tape is stretched or compressed, it will induce deformation to the sensing element, and the reflected optical signal will be modulated at the FPI accordingly.

For the experimental characterization, the sensing device extremities were attached to a fixed platform and a translation stage, as depicted in Figure 4b. The anchorage points were distanced 6 cm from each other. The theoretical characterization was performed by increasing the longitudinal elongation of the fiber from 0 up to 500 µm in a 20 μm step. The experimental characterization limits were set based on the theoretical analysis.

### 3.1. Theoretical Analysis

When a strain was applied in the optical fiber, it induced a small Δ*L* change in the FPI micro-cavity, *L*, which leads to a wavelength shift in the reflected interference modulated spectra, with *L* becoming *L* + Δ*L*, and *λ_m_* converting into *λ_m_* + Δ*λ_m_*_._ Therefore, and according to Equation (3), a longitudinal deformation (*ε_z_*) applied to the FPI micro-cavity will lead to a wavelength shift of the interference pattern according to [4]:

(6) $$\Delta \lambda =\frac{4L}{2m}\varepsilon_{z}$$

**Figure 5:**
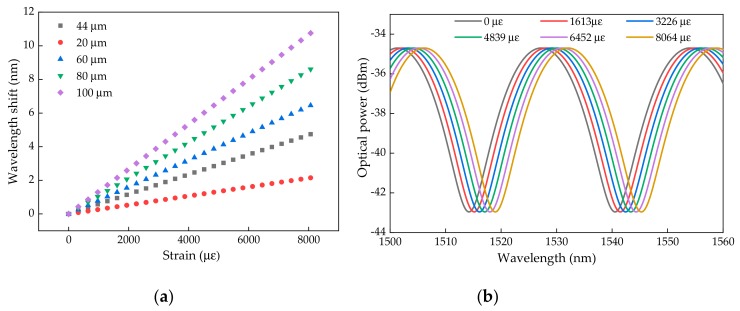
(**a**) Simulated dependency of the wavelength shift according to the applied strain for different cavity lengths (Equation (6)); (**b**) Simulated spectral function shift induced by different applied longitudinal elongations for a micro-cavity with a length of 44.07 μm (Equation (1)).

Figure 5a shows the simulated dependency of the wavelength shift (phase shift), according to the applied strain for different cavity lengths (Equation (6)). As expected, the sensitivity of the FPI micro-cavity is a function of the cavity length.

Considering a micro-cavity of 44.07 μm, as estimated for the cavity produced in this work, the phase shift induced by different applied strains can be modulated considering the spectral function given by Equation (1). As depicted in Figure 5b, the reflected optical spectrum moved to higher wavelengths with the applied strain.

**Figure 6:**
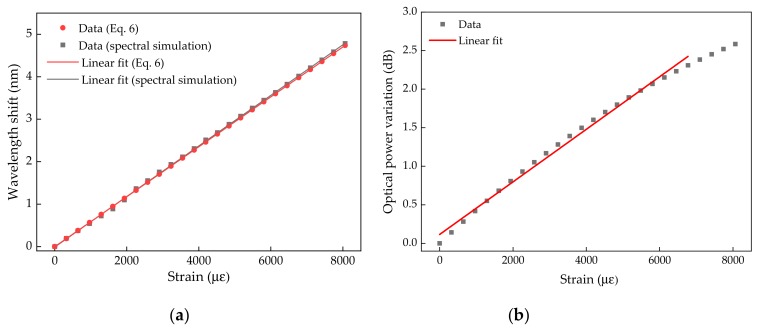
(**a**) Simulated wavelength shift as function of the applied strain considering Equations (1) and (6), and correspondent linear fit; (**b**) Simulated optical power variation as function of the applied strain, considering the integrated area from the convolution, given by Equation (5), and corresponding linear fit.

For the considered micro-cavity (*L* = 44.07 µm), and from the two theoretical approaches (analysis based on Equations (1) and (6)), we can assess the expected sensitivity of the proposed sensor, with respect to the wavelength shift as a function of the applied strain (Figure 6a), reaching a similar value for both approaches of 0.595 ± 0.002 pm/µε, with an *R^2^* of 0.9997.

When we considered the proposed interrogation approach, the sensor’s sensitivity evaluation was assessed in terms of the reflected optical power, given by the integrated area of the resulting convolution between the optical source and the FPI. Therefore, by applying Equation (5), and considering the optical source spectral function (*G*(*λ*)) and the FPI modulated spectral function for each applied strain (*I*(*λ_ε_*)), it is possible to estimate the optical power variation for each applied longitudinal elongation. In Figure 6b, the obtained theoretical results, regarding the optical variation as a function of the applied strain, are presented.

From the theoretically modulated results, displayed in Figure 6b, it is noticeable that the optical power dependency on the applied strain was not linear in the full range of the considered applied deformation. Therefore, a more thorough analysis on the linear range should be considered (range of action for the interrogation architecture proposed). It is obvious that the reflected optical power increased with the increase in the applied strain, nevertheless, its linearity range was dependent on the micro-cavity *FSR*. As shown earlier in Equation (4), the *FSR* corresponds to the wavelength distance between two consecutives maximums or minimums. As for the monitoring range of the proposed architecture, we need to consider that it is limited to less than 50% of the *FSR* value, where a linear dependency between the total reflected optical power and the FPI modulated signal is observed [10]. At the FPI transfer function maximum or minimum of interference, the optical power integrated area will be almost constant, and then it will start to change in the opposite direction. As smaller FPI micro-cavities present a higher *FSR*, they will provide a higher monitoring range for the interrogation architecture. Nevertheless, FPIs with lower *FSRs* will provide a higher optical power variation for the same value of wavelength shift, leading to better resolutions with a limited measuring range.

The FPI resolution is also dependent on the interference transfer function visibility (or fringe contrast), *V*. This parameter reflects the quality of the interference signal, which can be quantified by:

(7) $$V=\frac{I_{M}-I_{m}}{I_{M}+I_{m}}$$

where *I_M_* and *I_m_* are the maximum and minimum intensities of the fringes, respectively. The higher the fringe contrast (*V*), the higher the amplitude of the interference function of the FPI, which means that the spectral fringes are more defined, the signal to noise ratio is larger, and the accuracy and resolution measurements are better.

**Figure 7:**
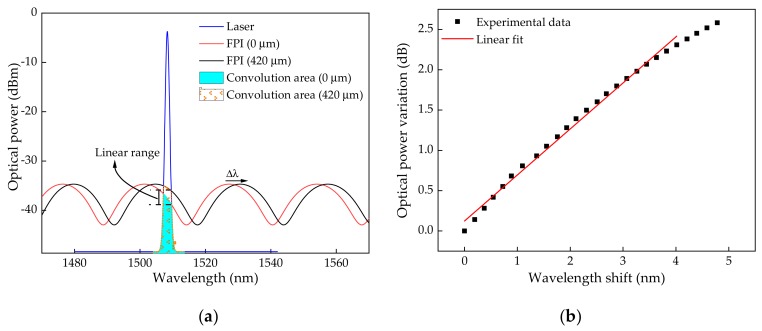
(**a**) Illustration of the proposed interrogation architecture limits; (**b**) Relationship between the FPI modulated transfer function wavelength shift and reflected optical power due to the laser and interference function convolution.

For the presented FPI micro-cavity, with an *L* = 44.07 µm and an *FSR* of ~27 nm (Figure 7a)), the interrogation range was <~13 nm, with a linear range of ~8 nm. Considering that the laser source peak is midway of the FPI linear region (~1508 nm in the range between ~1500–1514 nm of the FPI spectrum), it has a linear detection range of +4 nm for an increasing applied strain. Therefore, considering the linear range for the FPI in analysis, and taking into consideration the results displayed in Figure 6a, the monitoring range was limited to a deformation corresponding to 420 µm elongation, which induced a wavelength shift of ~4 nm of the FPI modulated transfer function. It should be noted that by reducing the application range down to 3 nm, corresponding to an applied elongation of ~320 μm, the linear range was more accentuated (with *R^2^* of 0.996). Nevertheless, the considered range up to 4 nm also presented a good linear fit. In the case of 0 to 420 µm of elongation, a sensitivity of (3.46 ± 0.06) × 10^−4^ dB/µε with a *R^2^* of 0.9928 was achieved (Figure 6b).

In Figure 7b, the relationship between the FPI modulated transfer function wavelength shift and reflected optical power due to the laser and interference function convolution, is plotted, leading to a dependency of 0.57 ± 0.01 dB/nm in the 0–420 µm longitudinal elongation range.

Therefore, from the theoretical analysis of the proposed architecture, the strain interrogation limit was set for this FPI sensing device, which was considered in the experimental implementation described in the following section.

### 3.2. Experimental Characterization

As previously mentioned, and considering the theoretical monitoring range limits, the experimental characterization of the developed sensing device was performed by applying an increasing strain, ranging from 0 up to ~7000 με (corresponding to an applied longitudinal elongation from 0 to 420 μm). It should be noted that this value range was only achieved because the FPI was encapsulated on the epoxy resin block.

For comparison and evaluation purposes, three different methodologies were used to analyze the optical signal modulated at the FPI location. First, for the validation of the theoretical approach applied in the previous section, an interrogation system (sm125, Micron Optics Inc., Atlanta, GA, USA) was used for the acquisition of the modulated spectrum for each elongation. The whole procedure was then repeated considering the proposed interrogation architecture for the optical power acquisition, first using a photodiode, and then in the second approach by using an OSA (Model FTB-500, EXFO, Quebec, Canada) instead of the photodiode. This equipment allows the spectral analysis of the convolution at the FPI, and provides the value corresponding to the total optical power within the convolution area.

**Figure 8:**
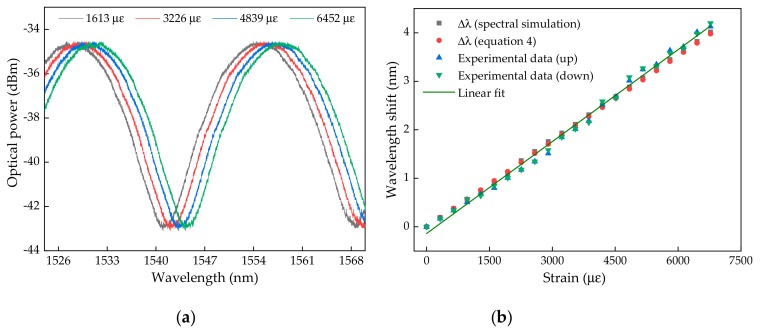
(**a**) Optical spectra acquired for 4 different elongation values; (**b**) Comparison of the wavelength shift obtained experimentally and theoretically as a function of the applied strain. The linear fit represented corresponds to the fit made at the “Experimental data (down)”.

Regarding the first set of tests comprising the spectral acquisition for each elongation applied on the optical fiber, the spectral feedback of the sensor is displayed in Figure 8a, for four different elongations. The tests were performed, initially with an increasing strain and followed right after with a decrease of the applied strain to evaluate the hysteresis of the sensing device. The wavelength shift for each longitudinal elongation up to 420 μm is presented in Figure 8b.

From the values displayed in the figure, the close similarity between the values obtained through the theoretical approach and the experimental data is clearly visible. Additionally, from the experimental data, for the increasing and decreasing strain, we achieved a strain sensitivity of 0.630 ± 0.010 pm/µε, which was close to the theoretical value (0.595 ± 0.002 pm/µε), and a maximum hysteresis of 0.070 nm.

**Figure 9:**
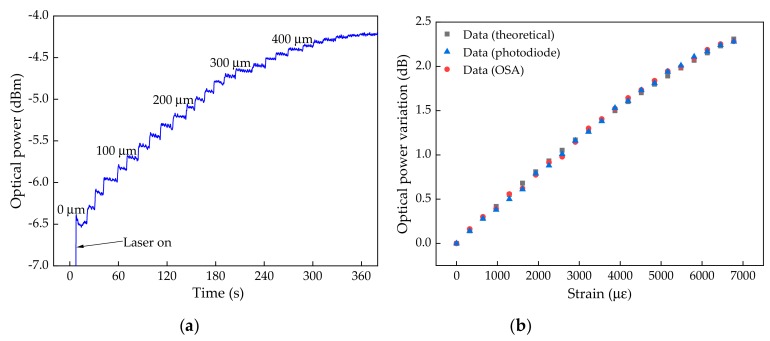
(**a**) Optical power values over a long time, obtained with the photodiode in the proposed architecture; (**b**) Optical power variation obtained theoretically and experimentally (photodiode and OSA) as a function of the strain values.

Regarding the second set of the experimental characterization, by using the proposed interrogation architecture, Figure 9a displays the optical power values along time, obtained with the photodiode. Each optical power step visible in the representation corresponded to an applied elongation. In Figure 9b, the optical power variation was plotted as a function of the strain applied for the data obtained using the photodiode in the proposed interrogation architecture as well as the experimental data corresponding to the total optical power variation in the scan range, acquired with the OSA. This acquisition was performed by replacing the photodiode in the proposed interrogation architecture, and acquiring a spectral information corresponding to each applied strain. For the presented experimental data acquired with the photodiode, a sensitivity of (3.47 ± 0.07) × 10^−4^ dB/µε and a resolution of 2 με were achieved.

The comparison between the theoretical data, considering the optical power given by the integrated convolution area of the FPI transfer function and the laser, and the acquired experimental data, is also visible in Figure 9b. It is clear that both data are in agreement, which validates the reliability of the proposed FPI interrogation architecture.

The theoretical values displayed in Figure 8b and Figure 9b were calculated considering a variation in *L*, assuming a uniform elongation. The similarity between these theoretical values and the experimental data reveals that the micro-cavity is also elongated at the same rate, despite its reduced cross section.

Regarding the acquisition rates, these were only dependent on the used photodiode and compliant acquisition software. The experimental data, presented in Figure 9a, were acquired at a rate of 250 Hz; nevertheless, the equipment used allows for the acquisition at rates up to 1 kHz, which is of high importance for some biomedical dynamic applications.

## 4. Overall FPI Sensing Architecture for Biomedical Applications

Aiming to validate the proposed interrogation architecture in applications requiring a high rate acquisition device, the developed sensing structure was tested in the monitoring of the carotid pulse wave form (CPW). This parameter reproduces the central hemodynamic conditions, providing vital information about the arterial health. Its analysis is one of the most used preventive measures, targeting the mitigation of cardiovascular episodes [11,12].

**Figure 10:**
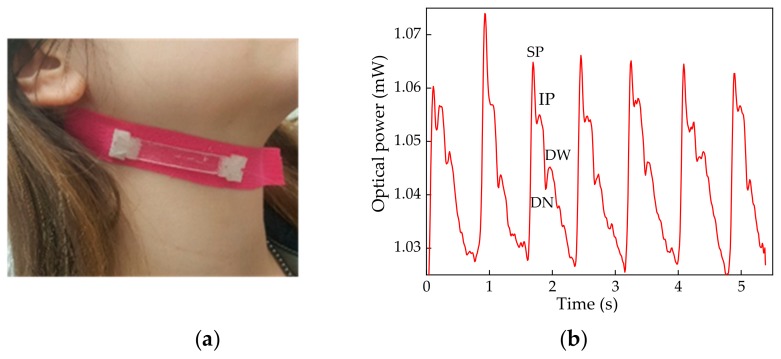
(**a**) Application of the biocompatible sensing block for carotid pulse wave monitoring; (**b**) Optical power variation according to the FPI modulation by the carotid pulse wave along the time.

To assess the performance of the proposed sensor and interrogation architecture in monitoring such vital parameters, the biocompatible elastic tape containing the sensing block was attached to a volunteer body at the carotid area, as depicted in Figure 10a. As the movement of the CWP extends/distends the elastic tape, it transfers such deformation to the sensing block as an applied strain, which modulates the FPI transfer function accordingly. Such modulation induces the reflected optical power to vary according to the typical carotid wave form, as depicted in Figure 10b.

The developed solution reveals itself to be a reliable standalone system to monitor CPW, allowing the acquisition of the carotid pulse waveform, where the waveform key points of analysis are clearly distinguished, namely the systolic pressure (SP), the inflection point (IF), the dicrotic notch (DC), and the dicrotic wave, as shown in Figure 10b.

Although the proposed sensor showed good performance as a standalone sensing device to monitor the CPW, further research should be presented in future work to target the optimization of the sensor design toward a smaller configuration as well as its validation, considering the performance comparison with other commercial or already validated devices. 

## 5. Conclusions

In this work, we proposed an alternative high rate acquisition interrogation architecture for FPI sensors. The close agreement between the experimental and the theoretical analysis proves the reliability of the overall architecture. It should be highlighted that within the micro-cavity design, certain issues should be taken into consideration including the required monitoring range limits, and the corresponding micro-cavity dimensions needed to achieve a linear dependency on the reflected optical power. We also verified that a trade-off between dynamic range and sensitivity is an inherent characteristic of the proposed interrogation scheme. Additionally, the FPI high visibility will enhance the interrogation slope and improve both the sensitivity and the system resolution.

Within the FPI micro-cavity used, an interrogation linear limit of 8 nm was achieved; nevertheless, due to the laser wavelength central peak at 1508 nm, the range was limited to 4 nm for a positive wavelength shift modulation. Within this linear range, the obtained sensitivities were (3.46 ± 0.06) × 10^−4^ dB/µε and (3.47 ± 0.07) × 10^−4^ dB/µε for the theoretical and experimental characterizations, respectively. Additionally, the preliminary results, targeting the application of the sensing device in biomedical scenarios, showed promising behavior in the standalone monitoring of the carotid pulse wave.
